# mTORC1 inhibitors: is temsirolimus in renal cancer telling us how they really work?

**DOI:** 10.1038/sj.bjc.6604636

**Published:** 2008-09-16

**Authors:** C Le Tourneau, S Faivre, M Serova, E Raymond

**Affiliations:** 1 Department of Medical Oncology, APHP and INSERM U728 (RayLab), Beaujon University Hospital, Clichy, France

**Keywords:** rapamycin, antiangiogenic, mTORC2, CCI-779, clear cell carcinoma

## Abstract

The proof of principle that a drug targeting mTOR can improve survival has been obtained recently from a large randomised trial using temsirolimus as a first-line therapy in patients with advanced poor prognostic renal cell carcinoma. Consistent data have recently shown the important role of the PI3K/AKT/mTOR signalling pathway in the regulation of crucial metabolic and mitotic functions of cancer cells and endothelial cells allowing a better understanding of the role of mTOR in controlling cancer cell proliferation and survival as well as tumour angiogenesis. As a result, rapamycin derivatives (rapalogues) that block mTOR/Raptor complex 1 were shown to exert direct antiproliferative effects against endometrial cancers, in which cancer cells frequently lose PTEN function as well as mantle cell lymphomas, in which cancer cell proliferation appears to be driven primarily by cyclin D1 overexpression. The overall antitumour effects of rapalogues in renal cell carcinoma appear to be more complex with tumour growth inhibition resulting from direct G1/S cell cycle blockage and/or apoptotic effects in carcinoma cells along with the inhibition of downstream signalling of the HIF1*α*-induced VEGF/VEGFR autocrine loop in endothelial cells shutting down the maintenance of tumour angiogenesis. Despite extensive cognitive researches, it is difficult to appraise which of those mechanisms is predominant in patients. This review focuses on mechanisms of action of rapalogues focusing on antitumour effects in patients with renal cell carcinoma.

Temsirolimus is the first-in-class mammalian target of rapamycin (mTOR) inhibitor that was approved for the treatment of patients with advanced poor prognosis renal cell carcinoma (Hudes *et al*, 2007). Mechanisms by which inhibition of mTOR may control tumour growth in patients with renal cancer remain unclear. Laboratory experiments have shown that antiproliferative effects of mTOR inhibitors in renal carcinoma cells may result from the inhibition of essential survival pathways, complex cell cycle effects, induction of apoptosis, and autophagy. Effects of mTOR inhibitors may also be anticipated in several other cellular target populations such as endothelial cells. The effect of mTOR inhibitors on angiogenesis is likely to have an important function in renal carcinoma, a highly vascularised tumour associated with a VHL-driven angiogenesis. Despite elucidating most of the effects of mTOR inhibitors in laboratory experiments, which of those potential mechanisms of action account for the most overall efficacy in patients, to what extent those effects are specific of renal tumour cell biology, and which of the multiple cell signal changes in cancer cells may serve as markers allowing better selection of tumour for efficacy is poorly known. In addition, dosing and schedules of mTOR inhibitors as well as drug metabolism and pharmacokinetics may somehow affect their biological effects and potential for combinability. In this brief review, we will attempt to address some of those issues based on currently published data with rapamycin derivatives (rapalogues).

## Brief Introduction to mTOR-Related Cell Signalling

**Figure 1:**
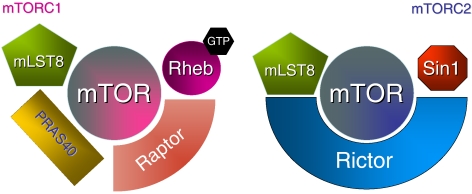
Mammalian target of rapamycin C1 (mTORC1) and mTORC2 multimolecular complexes.

**Figure 2:**
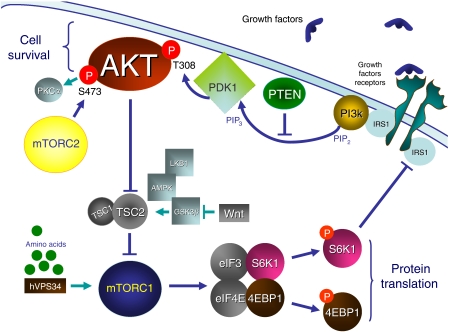
Cell signalling involving mTORC1 and mTORC2 in cancer cells and endothelial cells.

mTOR is a highly conserved serine/threonine kinase that forms multimolecular complexes and has a key function in apoptosis, cell growth, and tumour proliferation by controlling cellular catabolism and anabolism (extensively reviewed by Faivre *et al*, 2006a). mTOR may complex with raptor (regulatory-associated protein of mTOR) to form mTORC1, and can also complex with rictor (rapamycin-insensitive companion of mTOR) to form another multimolecular complex named mTORC2 (Figure 1). mTORC1 may eventually be activated by growth factors including the vascular endothelial growth factor receptor (VEGFR), the platelet-derived growth factor receptor (PDGFR), the epidermal growth factor receptor (EGFR), and the insulin growth factor receptor (IGFR), and nutrients through the phosphatidylinositol 3-kinase (PI3K) pathway (Figure 2). In the presence of nutrients, growth factors activate PI3K through the activation of receptor tyrosine kinases. Once activated, mTORC1 acts through its downstream effectors to stimulate protein synthesis and entrance into the G1 phase of the cell cycle through the eukaryotic translation initiation factor 4E-binding protein (4EBP1); the 40S ribosomal protein, p70 S6 kinase (S6K1); p27; cyclin D1; and proteins that regulate apoptosis including BAD, Bcl2, and p53. The PI3K/AKT/mTOR signalling pathway is under the control of PTEN, but it may also be inactivated by a feedback loop in which S6K1 directly inhibits IRS1. Mechanisms that trigger the activation of mTORC2 and the respective functions of mTORC1 *vs* mTORC2 in cancer cells remain unclear. Mammalian target of rapamycin C2 may also act upstream of mTORC1 by inducing the phosphorylation of AKT on serine 473, a mechanism that is thought to participate in cell survival and may account in resistance to rapalogues.

## Similarity and Differences between Rapalogues

Rapamycin (also named sirolimus, Wyeth) and other rapalogues including temsirolimus (CCI-779, Wyeth), everolimus (RAD001, Novartis Pharmaceutical), and deforolimus (AP23573, Ariad Pharmaceutical) are macrocyclic lactones acting as anticancer agents that target mTOR in several human cancers *in vitro* and *in vivo.* The main differences between rapalogues lie in changes in chemical properties in terms of drug solubility and metabolism. As a result, temsirolimus and deforolimus are water soluble and may be administered intravenously, whereas rapamycin and everolimus display low solubility, and therefore are available only for oral formulations. Rapalogues bind very similarly to the intracellular immunophilin-, FK506, binding protein-12 (FKBP12) and selectively inhibit mTORC1, but have no direct effects on mTORC2. Potency to inhibit mTORC1 seems to be identical across rapalogues. The inhibitory effects of rapalogues on mTORC1 do not seem to affect the kinase activity of mTOR. Although limited experiments have been carried out to benchmark and address cross-resistance between rapalogues, similarities in terms of chemical structures, mechanisms of action, affinity for the target, and overall spectrum of activity in laboratory experiments strongly suggest that currently developed rapalogues are similar in many ways, the main differences belonging to pharmacokinetic properties rather than to antitumor potency.

Inhibition of mTORC1 activity by rapalogues is reversible only slowly (about 5 days). Sensitivity and resistance to rapalogues may depend on the duration of drug exposure. Short exposure to rapalogues may result in the inhibition of mTORC1 that blocks the downstream S6K1 resulting in the inhibition of the S6K1 feedback loop, which in turn may help activate T308-AKT. For this reason, although mTORC1 is inhibited, mTORC2 may still remain efficient to activate S473-AKT and maintain cancer cell survival. Interestingly, sustained exposure to rapamycin was shown to secondarily inhibit mTORC2, as most of the mTOR bounded to rapamycin/FKBP12 is unavailable to complex with rictor. Those data may suggest that resistance to rapamycin may be associated with the activation of AKT, a mechanism that may be at least in part prevented using sustained exposure to rapamycin to block both mTORC1 and mTORC2. Thereby, antitumour activity may depend not only on the type of rapalogues and doses used in the clinic, but also on the duration of drug administration/exposure. Sustained exposure may increase the potency of rapalogues by inhibiting mTORC1 as well as mTORC2. Considering the half-life of rapalogues (see below), maximal mTOR inhibition may be achieved using continuous daily oral dosing of everolimus, whereas temsirolimus that is slowly biotransformed into sirolimus can be given intravenously only once a week.

Temsirolimus was the first mTORC1 inhibitor investigated in clinical trials in the late 1990s in patients with cancer. Temsirolimus given intravenously on a weekly schedule showed a safe toxicity profile, the most prevalent toxicities being reversible skin toxicity, stomatitis, and thrombocytopenia. Pharmacokinetic analysis showed that temsirolimus was converted into sirolimus, and exposure to sirolimus was prevalent in plasma several days after a single infusion of temsirolimus (Raymond *et al*, 2004; Hidalgo *et al*, 2006). The respective roles of sirolimus and CCI-779 in antitumor effects of temsirolimus are yet to be proved. Everolimus has subsequently been tested orally either on weekly or daily schedules. Everolimus was also well tolerated, skin toxicity being the most prevalent side effect. Similar results were obtained with deforolimus. For those agents, dose-limiting toxicity was not reached, and doses recommended for phase II studies were based on compromises between side effects, pharmacokinetic, and pharmacodynamic data. In addition, considering the long pharmacokinetic and biological half-lives of those agents, the advantages of daily *vs* weekly schedules remain unclear. Although those drugs were somehow different, daily doses associated with the antitumour effects of rapalogues ranged between 10–25 mg, whereas weekly doses recommended for phase II studies were ⩾25 mg.

## Pharmacokinetic Limitations of Rapalogues

Data specifically investigating the oral absorption and biodisponibility of oral rapalogues primarily derive from those of sirolimus. Recent data have shown that absorption of oral rapalogues may be limited and participate in interpatient variability (O'Donnell *et al*, 2008). Patient oral bioavailability may be dependent on the expression of ATP-binding cassette membrane transporters in the gut accounting for variability in plasma concentrations and exposures using current dosing of everolimus. Temsirolimus as well as other rapalogues are metabolised primarily in the liver by the cytochrome CYP450 3A4/5. Pharmacokinetic studies showed that everolimus and deforolimus do not require biotransformation for activity and no major metabolite was reported. Conversely, temsirolimus displays a complex metabolism as the parental drug CCI-779 is rapidly cleared from the plasma and converted by CYP3A4 into sirolimus that becomes the most prevalent species after temsirolimus intravenous infusion, and sustains at relatively high concentrations for several days (Raymond *et al*, 2004). Bioconversions of CCI-779 into sirolimus appear to be less than dose proportional, suggesting a saturation of the CYP3A4 capacity at higher dosing (Raymond *et al*, 2004). In addition, exposure to deferolimus appears to be less than dose proportional (Mita *et al*, 2008). As both CCI-779 and sirolimus display similar effects on mTORC1, the respective roles of the parental drug and its main metabolite sirolimus on the overall activity of temsirolimus in renal cancer remain unknown. Pharmacokinetic data demonstrated that exposure to rapalogues is strongly increased by the concomitant administration of drugs that are substrates, activators, and inhibitors of CYP3A4 such as rifampicin, anticonvulsants, and immunosuppressive drugs such as cyclosporine (Boni *et al*, 2007; Kuhn *et al*, 2007).

## Differential Review of Biological Effects of mTOR Inhibition in Cancer Cells

### Do rapalogues induce direct antiproliferative effects through cell cycle arrest, apoptosis, and autophagy in renal carcinoma cells?

Rapalogues may exert antitumour effects by inducing dose-dependent cell cycle arrest, apoptosis, and autophagy in cancer cells. Direct antitumour effects of mTORC1 inhibitors may require specific biological conditions including the activation of the PI3K/AKT/mTORC1 signalling as well as functional apoptotic pathways, and the circumstances that render cancer cells are highly vulnerable to rapalogues (Faivre *et al*, 2006a). Whether these mechanisms have a function in the clinical activity of rapalogues in renal cell carcinomas remain unknown. Several clear cell carcinoma cell lines express high AKT levels and reduced PTEN expressions that render them potentially sensitive to mTOR inhibition (Hara *et al*, 2005). Activity of mTORC1 inhibitors, particularly temsirolimus, in renal cell cancer has raised the possibility that responders share a common molecular phenotype that renders these tumours dependent on mTOR for growth and/or survival. In renal cell cancer, *PTEN* gene expression has been shown to be downregulated in a large percentage of cases, presumably by epigenetic silencing (Brenner *et al*, 2002; Velickovic *et al*, 2002). In particular, lack of PTEN expression has been shown to be an independent negative prognostic factor for disease-specific survival in patients with metastatic renal cell carcinoma (Kim *et al*, 2005). However, in culture, most of the renal cancer cell lines remain poorly sensitive to rapalogues. In our experience, concentrations that may be reached in clinical trials are associated only with mild antiproliferative effects with accumulation of cells in late G1 of the cell cycle (Raymond E, personal communication). Concentrations leading to apoptosis and autophagy are observed only at high concentrations barely compatible with pharmacokinetic exposures that may be reached in the patients. To our knowledge, tumour types highly vulnerable to rapalogues include endometrial cancer (Kanamori *et al*, 2001; Uegaki *et al*, 2005; Oza *et al*, 2006; Colombo *et al*, 2007; Milam *et al*, 2007) that frequently displays PTEN loss of function and mantle cell lymphoma (Grewe *et al*, 1999; Witzig *et al*, 2005; Ansell *et al*, 2006) that overexpress cyclin D1. The effects of rapalogues in mantle cell carcinoma are consistent with preclinical studies showing that mTORC1 inhibitors could downregulate cyclin D1 (Aguirre *et al*, 2004). In those two examples, the susceptibility of vulnerable endometrial cancer and mantle cell lymphoma cells to rapalogues was obviously not dose-dependent. Conversely, on the basis of the current knowledge, it is unlikely that renal carcinoma cells display a more similar vulnerability than endometrial cancer and mantle cell lymphoma to temsirolimus or other rapalogues. In preclinical models, renal carcinoma cells appeared to be poorly susceptible to rapalogues, and most of the antiproliferative effects were dose-dependent, occurring at concentrations that may have been far beyond those achievable in the clinic. In patients with renal cell carcinoma, several doses of temsirolimus have been tested in clinical trials but toxicity leads to refrainment from using the highest possible dosing. As a result, it is difficult to believe that the currently recommended doses of temsirolimus (and resulting plasma exposures) are high enough to yield direct effects in renal carcinoma cells, that is, to induce potent cell cycle inhibition and/or cell death induction (either by apoptosis or autophagy). If a direct effect of rapalogues against renal cancer cells is unlikely, temsirolimus may induce antitumour effects by targeting other cells with activated mTOR signalling such as the cells participating to tumour angiogenesis.

### Do the effects of rapalogues in renal cancer rely on antiangiogenic properties?

Renal cell carcinoma is acknowledged to be an abundantly vascularised tumour, in which cancer cells are primarily resistant to chemotherapy-induced apoptosis. Historically, treatment for patients with metastatic renal cell carcinoma has been limited. Standard chemotherapeutic agents have been ineffective and cytokine-based treatment with interleukin 2 or interferon-*α* benefited only ⩽10–20% of patients. Initially, the phase I studies of rapalogues showed objective responses in several patients with renal cell carcinomas (Raymond *et al*, 2004; Hidalgo *et al*, 2006).

On the basis of exciting results observed in phase I trials, intravenous weekly temsirolimus was investigated in a large dose-randomised (25, 75, or 250 mg) phase II study of patients with advanced renal cell carcinoma refractory to cytokine-based therapy who were classified into three groups according to Motzer's criteria (Atkins *et al*, 2004). Overall, 85% of patients had received prior interleukin 2 and 45% interferon-*α* treatment. As a single agent, temsirolimus displayed a relatively low objective response rate of 7%, with 26% additional minor responses (Table 1). Interestingly, 17% of patients had stable disease lasting more than 6 months. Higher survival rate was observed in patients with Motzer's intermediate or poor prognosis criteria who experienced a nearly twofold increase in survival (22.5 months for the intermediate group and 8.2 months for the group with poor prognosis) compared with historical controls treated with interferon-*α* (13.8 and 4.9 months for the intermediate and poor prognosis groups, respectively). Interestingly, neither toxicity nor efficacy was significantly influenced by the temsirolimus dose level. Furthermore, a temsirolimus dose of 25 mg weekly was selected for future exploration. These promising results subsequently led to the initiation of a large multicentre randomised phase III trial comparing interferon-*α* given either alone or with temsirolimus, or a single agent weekly intravenous administration of 25 mg temsirolimus as a first-line treatment in a total of 626 high-risk patients according to Motzer's criteria with advanced or metastatic renal cell carcinoma (Hudes *et al*, 2007). The trial showed that patients treated with temsirolimus had a statistically significantly longer median survival than those receiving interferon-*α* (10.9 *vs* 7.3 months, *P*=0.008). Interestingly, the combination of temsirolimus with interferon-*α* did not improve survival in those patients. This study was the first clinical trial showing a survival benefit in using an inhibitor of mTOR in patients with cancer. Promising results have also been reported with everolimus in 41 patients with advanced renal cell carcinomas who had received up to one prior therapy (Jac *et al*, 2007). Everolimus was given at a daily dose of 10 mg orally without interruption. The objective response rate was 32%.

**Figure 3:**
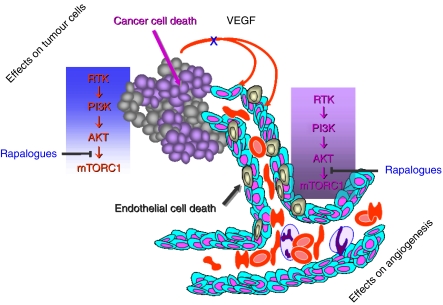
Function of the PI3K/AKT/mTOR signalling in cancer. Mammalian target of rapamycin (mTOR) may be activated in cancer cells and endothelial cells and may participate in cancer cell proliferation and tumour angiogenesis. Rapalogues that block downstream activation of this pathway may induce direct antiproliferative effects in cancer cells and may eventually inhibit tumour angiogenesis.

A weekly intravenous dose of temsirolimus induces sustained exposure to low plasma concentrations of sirolimus, whereas 10 mg daily dose of everolimus achieves low plasma concentration of this drug. What makes renal tumours so sensitive to low dose/low exposure of mTORC1 inhibitors if renal cancer cells are not so intrinsically sensitive? Preclinical data, as well as the great efficacy of antiangiogenic therapies targeting VEGFR and PDGFR such as sorafenib and sunitinib in renal cell cancer (Faivre *et al*, 2006b, 2007; Escudier *et al*, 2007; Motzer *et al*, 2007b), suggested that mTORC1 inhibitors may also have antiangiogenic properties in renal cell cancer. Indeed, it has been shown that mTORC1 inhibitors can target tumour growth indirectly, by interacting with the maintenance of endothelial cells and pericytes that are required for tumour angiogenesis. Tumour angiogenesis relies on an intricate interplay between tumour cells, endothelial cells, and surrounding mesenchymal cells (pericytes in microvessels and vascular smooth-muscle cells in large vessels) to activate endothelial cell proliferation, to recruit migrating endothelial cells and pericytes, and to form new vessels through vascular remodelling and maturation (Hamada *et al*, 2005). At the molecular level, tumour angiogenesis depends on vascular growth factors such as VEGF, PDGF, basic fibroblast growth factor (bFGF), and members of the tumour growth factor-*β* (TGF*β*) superfamily. Interestingly, all the above factors have been shown to be able to activate the PI3K/AKT/mTOR pathway in cancer cells, endothelial cells, or pericytes (Guba *et al*, 2002). Cellular proliferation, survival, and migration required for vascular sprouting, as well as endothelial cell differentiation, lead to tubule formation that is driven primarily by VEGF/VEGFR activation, which can in turn trigger the PI3K/AKT/mTOR pathway (Figure 3). One of the major stimuli of cancer angiogenesis is hypoxia, which activates hypoxia-inducible transcription factors (HIFs), which in turn induce the expression of VEGF, VEGFR, bFGF, and PDGF. Mammalian target of rapamycin can facilitate the translation of HIF1*α* mRNA, thereby enhancing the vascular growth factor expression (Arsham *et al*, 2002). In normal vessels, HIF1*α* is transiently expressed as a result of the action of the HIF-prolyl hydroxylase that targets HIF1*α* to a ubiquitin ligase complex containing von-Hippel–Lindau (VHL), which marks it for destruction by the proteasome. In renal cancer, loss-of-function mutations of VHL can cause HIF1*α* stabilisation, thereby inducing VEGF and PDGF overexpression and sustained tumour angiogenesis (Le Tourneau *et al*, 2007). Inhibition of mTORC1 by temsirolimus has been shown to reduce expression of HIF1*α* and HIF2*α* under both normoxic and hypoxic conditions in mouse xenograft models (Thomas *et al*, 2006). In addition, recent work has shown that temsirolimus preferentially inhibits VHL-null renal cell carcinomas (Thomas *et al*, 2006). On the basis of pharmacokinetic data, those effects may be expected in the clinic at doses of rapalogues above 20–25 mg per week. The observed clinical efficacy of mTORC1 inhibitors in patients with renal cell carcinoma may be mediated in part by dependence of efficient HIF translation on the mTOR pathway by intercepting the VEGF/VEGFR and/or PDGF/PDGFR signalling cascades. Overall, those data strongly suggest that the anticancer effects of mTORC1 inhibitors involve antiangiogenic processes mediated by effects on endothelial cells and pericytes rather than on renal carcinoma cells themselves.

## Usefulness of Surrogate Markers and Imaging to Monitor the Effects of Rapalogues

Monitoring the biological activity of rapalogues to determine the biologically active dose rapidly appears as a challenge in patients participating in clinical trials. As rapalogues interfere with glucose metabolism using mechanism independent of their antitumour effects, 18-FDG PET scan appears inappropriate to reliably monitor the effects of rapalogues (O'Donnell *et al*, 2008). Surprisingly, the effects of rapalogues on the levels of cholesterol and triglyceride were never fully considered to monitor the biological effects of rapalogues in trials in patients with cancer. As easily available cells were peripheral blood mononuclear cells (PBMCs), most studies were carried out looking at the phosphorylation of S6K and 4EBP1 in those cells (Hidalgo *et al*, 2006; Mita *et al*, 2008; O'Donnell *et al*, 2008; Tanaka *et al*, 2008). Those data consistently showed a dose-dependent effect of rapalogues in inducing a dephosphorylation of S6K and/or 4EBP1 in PBMC. In a recent study, we showed that modelling the S6K dephosphorylation in PBMC in animals and humans under exposure to everolimus allows prediction of a threshold level of activity of this drug in humans and helps select dosing for phase II studies (Tanaka *et al*, 2008). Similarly, another study addressed the direct effects of everolimus on phosphorylation of several kinases including S6K and AKT in skin and tumour tissue biopsies (Tabernero *et al*, 2008). The authors showed that exposure to continuous everolimus more readily induced inhibition of S6K phosporylation and activation of S473-AKT at doses above 10 mg per day and 50 mg per week. Unfortunately, because of the limited number of patients entered in those studies, no correlation was made between molecular changes, toxicity, and activity of everolimus. Thus, it is unclear whether these molecular markers are reliable for addressing the efficacy of rapalogues in tumour or whether they may reflect only the molecular changes on drug translation induced by rapalogues. Furthermore, none of the currently tested biomarkers reliably addressed the antiangiogenic effects of rapalogues and, for instance, little is known of the effects of rapalogues on VEGF and sVEGFR2 in plasma of patients treated for renal cell carcinomas. As no clear correlation between antiproliferative/antitumor effects and inhibition of phosphorylations of S6K and 4EBP1 has been demonstrated, dose recommendations that are based on currently studied surrogate biomarkers may be underestimated. In our opinion, dose recommendations for current and new rapalogues shall still be made on the basis of evaluation of toxicity.

## The Limitation of Rapalogues in Monotherapy and the Potential for Combination Therapy

Although rapalogues have displayed activity in a number of malignancies, the antitumour effects of rapalogues in monotherapy appear to be limited, the primary and acquired resistances to mTORC1 inhibition being observed in the vast majority of tumour types. Thereby, combinations seeking to broaden the spectrum of activity and overcome resistance to rapalogues have been investigated. The PI3K/AKT/mTOR signalling pathway has an important function in cancer cell survival in response to the insult induced by cytotoxic agents. Key factors for primary resistance may involve survival and apoptosis signalling pathways. First, tumour cells may be able to maintain survival and proliferation by using redundant cross-signalling pathways involving particularly MAPK. Second, tumour cells may have non-functional apoptotic pathways, especially when expressing Bcl2 (Aguirre *et al*, 2004). When survival and apoptotic signalling pathways are involved in resistance to rapamycin, one solution to overcome resistance may be based on combinations with other anticancer agents, especially potent apoptosis inducers.

Mammalian target of rapamycin C1 inhibitor combinations with conventional chemotherapy were designed to enhance the proapoptotic effects of rapalogues with cytotoxics and to broaden the spectrum of activity in tumour types only marginally sensitive to single-agent rapalogues. However, combinations of rapalogues with 5-fluorouracil and gemcitabine were associated with toxicities barely compatible with clinical applications (Faivre *et al*, 2006a). Furthermore, this approach may be of little relevance in renal cell carcinoma for which no cytotoxic agent showed any activity.

The other possibility is to combine mTORC1 inhibitors with targeted agents to avoid resistance mechanisms related to survival signalling pathways. The goal of this strategy is either to optimise inhibition of a pathway involving PI3K/AKT/mTOR or to inhibit multiple signalling pathways. Strategy may consist, for example, in combining an mTORC1 inhibitor with a tyrosine kinase receptor inhibitor. Recent preclinical studies combining rapamycin with inhibitors of EGFR or KIT receptors have shown synergistic effects (Johnson *et al*, 2007). A number of combinations are currently investigated using rapalogues with targeted agents. For example, the combination of EGFR inhibitors with rapalogues offer a strong potential for further clinical investigations in tumour type responding to EGFR inhibitors such as lung and head-and-neck carcinomas (Bianco *et al*, 2008). Whether those combinations may have a potential in renal cell carcinoma remains to be investigated.

Finally, another possibility may consist, for example, in combining an mTORC1 inhibitor with drugs targeting angiogenesis. The antiangiogenic properties of both interferon-*α* and temsirolimus, along with their activity as a single agent in renal cell carcinoma provided a strong rationale for investigating their combinations in clinical trials. Phase I/II trials showed that the combination of temsirolimus with interferon was feasible with a safe toxicity profile (Motzer *et al*, 2007a). Surprisingly, survival of the combination of temsirolimus with interferon-*α* was not superior to temsirolimus given as a single agent (Hudes *et al*, 2007). Other antiangiogenic agents such as bevacizumab, sorafenib, and sunitinib recently showed potent efficacy in patients with renal cell carcinoma. Combinations of temsirolimus with sorafenib and sunitinib in clinical trials are anticipated to be associated with possible pharmacokinetic interactions as those agents are expected to be catabolized by the same cytochromes in the liver. Conversely, recent unpublished data suggest that combining bevacizumab with temsirolimus is feasible and may have clinical potential to enhance survival of patients with advanced kidney cancer.

## ‘Non-Rapalogues’ mTOR Kinase Inhibitors

In this study, we stressed that some tumour types with abnormalities involving the PI3K/AKT/mTOR pathway or with sustained antiangiogenic activity such as kidney cancers are highly sensitive to mTORC1 inhibitors. Nevertheless, most tumour types are only marginally sensitive to mTORC1 inhibitors either because of redundant signal transduction pathways, lack of functional apoptosis/autophagy, or expression of VEGF/VEGFR-independent angiogenesis. Furthermore, although acute toxicity of rapalogues is fairly acceptable, long-term exposure to several rapalogues is associated with interstitial pneumonitis (Vahid and Marik, 2008) that may require treatment discontinuation and jeopardise efficacy. It is thus important to consider other drugs blocking the mTOR signalling pathways by different mechanisms of action with distinct toxicity and overcoming current mechanisms of resistance to rapalogues.

One of the key factors that might be involved in primary resistance to mTORC1 inhibitors is the target itself. The rapamycin-insensitive complex mTORC2 may limit the efficacy of rapalogues by activating AKT. AKT activation may stimulate several survival pathways that stimulate proliferation and inhibit apoptosis. In this case, it might be interesting to investigate the new generation of mTOR inhibitors that are designed to block the kinase of mTOR, thereby inhibiting both mTORC1 and mTORC2. Drugs (some developed by Astra-Zeneca and Celgene) that target the mTOR kinase are expected to be non-cross resistant to rapamycin and to broaden the spectrum of activity of current rapalogues. Acute and long-term toxicity, and combinability may also be different from that of rapalogues. Mammalian target of rapamycin kinase inhibitors are currently in preclinical development and may soon enter clinical trials.

## Conclusions

Rapalogues inhibiting mainly mTORC1 have shown pleiotropic effects targeting both cell proliferation and survival, ultimately leading to antiproliferative and antiangiogenic effects. In renal cell cancer, which appears as an example of a tumour sensitive to temsirolimus, the inhibition of angiogenesis presumably represents one of the major mechanisms of antitumour effects. Future studies should aim at identifying biological parameters that may predict the antitumour activity of mTORC1 inhibitors, distinguishing which of the possible mechanisms, that is, cell cycle blockage, apoptosis, and autophagic induction in cancer cells *vs* antiangiogenic effects, are predominant. Inhibition of the kinase of mTOR represents an interesting new approach to target signalling of mTORC1 and mTORC2.

## Figures and Tables

**Table 1:** Clinical results of rapalogues in patients with renal cell carcinoma

**Compound**	**Status**	**Tumour type and setting**	**Dose and schedule (no of patients)**	**Clinical results**	**References**
Temsirolimus	Phase I	All tumours	7.5–220 mg per week i.v. (*n*=24)	Objective response in interferon-*α* and interleukin 2 refractory renal cell carcinoma	Raymond *et al* (2004)
Temsirolimus	Phase II	R/M RCC refractory to cytokine-based therapy	25 vs 75 *vs* 250 mg per week i.v. (*n*=111)	ORR: 7% with a nearly twofold survival improvement for intermediate/poor prognosis patients with historical series	Atkins *et al* (2004)
Temsirolimus	Phase III	First-line R/M RCC	25 mg per week i.v. (*n*=626)	Significantly longer survival in temsirolimus arm (10.9 months) compared with interferon-*α* (7.3 months)	Hudes *et al* (2007)
Temsirolimus+ interferon *α*	Phase I/II	R/M RCC mainly refractory to cytokine-based therapy	5–25 mg per week i.v. temsirolimus with 6 MU interferon-*α* (*n*=71)	ORR: 8% with 36% of patients having tumour stabilisation of more than 24 weeks	Motzer *et al* (2007a)
Everolimus	Phase II	First- or second-line R/M RCC	10 mg daily orally (*n*=41)	Response rate=: 32% Stable disease>3 months: 51%	Jac *et al* (2007)

i.v.=intravenous; ORR=overall response rate according to RECIST criteria; RCC=renal cell cancer; R/M=recurrent or metastatic.
